# The Effects of Implicit and Explicit Motor Learning in Gait Rehabilitation of People After Stroke: Protocol for a Randomized Controlled Trial

**DOI:** 10.2196/resprot.9595

**Published:** 2018-05-24

**Authors:** Li-Juan Jie, Melanie Kleynen, Kenneth Meijer, Anna Beurskens, Susy Braun

**Affiliations:** ^1^ Research Center of Nutrition, Lifestyle and Exercise Faculty of Health Heerlen Netherlands; ^2^ Caphri School for Public Health and Primary care Maastricht University Maastricht Netherlands; ^3^ Nutrim School for Nutrition and Translational Research in Metabolism Department of Nutrition and Movement Sciences Maastricht University Maastricht Netherlands; ^4^ Research Centre for Autonomy and Participation of Persons with a Chronic Illness Zuyd University of Applied Sciences Heerlen Netherlands

**Keywords:** motor learning, implicit learning, explicit learning, analogy, analogy learning, gait, physiotherapy, rehabilitation, stroke, CVA

## Abstract

**Background:**

A significant part of neurological rehabilitation focuses on facilitating the learning of motor skills. Training can adopt either (more) explicit or (more) implicit forms of motor learning. Gait is one of the most practiced motor skills within rehabilitation in people after stroke because it is an important criterion for discharge and requirement for functioning at home.

**Objective:**

The aim of this study was to describe the design of a randomized controlled study assessing the effects of implicit motor learning compared with the explicit motor learning in gait rehabilitation of people suffering from stroke.

**Methods:**

The study adopts a randomized, controlled, single-blinded study design. People after stroke will be eligible for participation when they are in the chronic stage of recovery (>6 months after stroke), would like to improve walking performance, have a slow walking speed (<1 m/s), can communicate in Dutch, and complete a 3-stage command. People will be excluded if they cannot walk a minimum of 10 m or have other additional impairments that (severely) influence gait. Participants will receive 9 gait-training sessions over a 3-week period and will be randomly allocated to an implicit or explicit group. Therapists are aware of the intervention they provide, and the assessors are blind to the intervention participants receive. Outcome will be assessed at baseline (T0), directly after the intervention (T1), and after 1 month (T2). The primary outcome parameter is walking velocity. Walking performance will be assessed with the 10-meter walking test, Dynamic Gait Index, and while performing a secondary task (dual task). Self-reported measures are the Movement Specific Reinvestment Scale, verbal protocol, Stroke and Aphasia Quality of Life Scale, and the Global Perceived Effect scale. A process evaluation will take place to identify how the therapy was perceived and identify factors that may have influenced the effectiveness of the intervention. Repeated measures analyses will be conducted to determine significant and clinical relevant differences between groups and over time.

**Results:**

Data collection is currently ongoing and results are expected in 2019.

**Conclusions:**

The relevance of the study as well as the advantages and disadvantages of several aspects of the chosen design are discussed, for example, the personalized approach and choice of measurements.

**Trial Registration:**

Netherlands Trial Register NTR6272; http://www.trialregister.nl/trialreg/admin/rctview.asp?TC=6272 (Archived by WebCite http://www.webcitation.org/6ytA937m5)

**Registered Report Identifier:**

RR1-10.2196/9595

## Introduction

### Background and Rationale

For most people, walking is a motor skill that generally takes place without too much effort. However, for people who have suffered a stroke, walking is often suddenly impaired, which can lead to major consequences in daily life functioning. People may experience impaired walking patterns with lower walking speeds, which has been associated with lower levels of functional ambulation [1]. Gait training is one of the main components of physiotherapy within stroke rehabilitation because it is an important criterion for discharge and requirement for functioning at home [1,2]. Evidence suggests that even in later (chronic) stages after stroke, people are still able to improve motor performance [3]. Many different techniques and therapies can be used to improve walking performance [4]; the challenge for physiotherapists is to choose and deliver gait training in the most efficient and effective manner. Moreover, preferably, obtained improvements in performance are durable over a longer period and resilient under different circumstances and in dual-task situations, for example, walking and talking. Despite the availability of new training approaches such as the use of robotics [5], virtual reality, for example through exergames [6,7] or body weight support training [8], overground walking [9] still seems one of the most applied gait-training approaches in clinical practice. The current literature and clinical guidelines encourage the use of context- and task-specific treatment approaches, an example of which is overground walking [4,10].

To apply overground gait training, physiotherapists are encouraged to use general motor learning principles [10]. Within the context of motor learning, a broad distinction between implicit and explicit motor learning has been described [11,12]. Explicit motor learning can be defined as learning generated by verbal knowledge of movement performance; it involves cognitive stages within the learning process and is dependent on working memory involvement [13]. The definition indicates that the learner is aware of all the underlying facts and rules of the to-be-learned motor skill during the process of learning. In practice, verbal explicit instructions are frequently used, and often these instructions encourage patients to be aware of their own body movements [14]. For example, in gait training, therapists tell patients to think about their performance, for example, “Move your hips to the left and straighten your knee before stepping” [14].

In contrast to explicit learning, implicit motor learning progresses with no or minimal increase in the verbal knowledge of movement performance (eg, facts and rules) and without awareness [13]. Learning is suggested to take place more automatically and in a less conscious manner. The learner is aware of the process of learning but cannot recall the underlying facts and rules of the motor skill. Gait training could be facilitated more implicitly, for example, when a physiotherapist would gradually constrain or change the environment, for example, when letting the person walk over different surfaces. In this situation, verbal instructions are not needed, but the environment facilitates the motor skill (walking). An observational study demonstrated that often multiple learning strategies are being used within one training session [15]. These different learning strategies may represent an implicit-explicit continuum on which some promote more implicit and others more explicit forms of learning [11]. Looking at the current practice, therapists seem to have a preference for learning approaches that are related to explicit learning in which often high numbers of verbal explicit instructions are used [14].

Various advantages of implicit motor learning over explicit motor learning have been reported in literature [12]. Studies have demonstrated that individuals who learned motor skills implicitly perform the motor skill better under pressure, perform better in dual-task situations, and perform better over time compared with their explicit counterparts [12,16,17]. Although these studies have primarily been conducted within the healthy population, implicit learning may also be advantageous for the patient population [18,19]. For example, implicit motor learning may be of extra benefit to those with cognitive deficits [20]. Reduced cognitive function is frequently seen in people after stroke [21] and it often hampers the process of motor learning. The degree by which these cognitive functions are being evoked can be influenced by the choice of learning approaches [20]. An interesting feature of implicit motor learning is the assumption that it is less reliant on working memory resources, that is, it involves less cognitive functions, compared with explicit motor learning [19]. It is, therefore, intriguing to explore the effects of implicit motor learning within the stroke population.

Although from a theoretical perspective, the features of implicit motor learning have been described, its practical application in clinical practice seems more complex. Various learning strategies, for example, dual task, or errorless learning have been shown to promote implicit motor learning [17,22]. One learning approach that may also be placed more on the implicit side of the implicit-explicit continuum is analogy learning. In analogy learning, the learner is provided with one single analogy or metaphor that strives to combine all the relevant rules of the to-be-learned motor skill. Early studies on analogy learning took place within a sporting context, for example, to learn specific skill techniques in table tennis or basketball [23,24]. A good example in this regard was presented by Lam and colleagues [23]. They used the analogy instruction “Shoot as if you are trying to put cookies into a cookie jar on a high shelf” to teach basketball players to impart backspin on the basketball. At present, there seems an increased interest of its application within different contexts. Analogy studies have been performed with older people [25] in the context of speech therapy [26] and within neurological populations [27,28]. For example, the analogy “imagine as if you are walking over a frozen lake” has been used in gait rehabilitation to facilitate lifting and placing the foot while walking [28]. With regard to gait rehabilitation, small pilot studies have reported that analogies can be used in a feasible manner to facilitate walking performance [27,28]. It has been reported that the analogy should lead to the desired biomechanical movement and that preferably the analogy should contain a meaningful component to the participant [28,29]. In addition to feasibility, trends toward improved walking performance have been observed following analogy interventions, which demonstrates the potential of analogy learning in clinical practice [27,28]. However, to further establish the effectiveness of analogy learning in clinical gait rehabilitation, larger sample sizes and research designs using a control condition are required.

This study describes the design for a randomized controlled study to assess the effects of implicit motor learning compared with explicit motor learning on walking speed in people suffering from stroke. The concept of analogy learning is used to structure the gait-training sessions within the implicit condition, whereas explicit motor learning is promoted by using extensive verbal instructions and feedback. A process evaluation is embedded to investigate feasibility and fidelity of the applied interventions.

### Research Question

The following research question was established to examine the effects of implicit and explicit motor learning in gait rehabilitation of people after stroke: Is a 3-week implicit motor learning walking intervention (analogies) more effective compared with a 3-week explicit motor learning walking intervention (verbal detailed instructions) delivered at home with regard to walking speed in people suffering from stroke?

## Methods

### Study Design

The study adopts a randomized, controlled, single-blinded study design in people suffering from stroke in the chronic stage of recovery. The study has been approved by the local ethics committee METC-Z in Heerlen, the Netherlands (NL number: NL.60338.096.16, Ethics nr: 17-T-06). After baseline measures, eligible participants will be randomized to the implicit or explicit condition (T0). Outcome assessments will take place directly after the intervention (T1) and again one month later (T2).

### Involvement of Client Representatives

Throughout the design and planning of the study, 3 patient representatives were involved in every step of the decision-making process regarding the design and execution of the study. In several consensus meetings, they represented the patient’s perspective, particularly with regard to the feasibility aspects of the study design. They were also involved in customizing participant information letters and promotion material for the study.

### Population

The study population consists of people who had a stroke and who are living at home. People will be recruited via local private practices, rehabilitation institutes, and a local health-related newspaper. Participants will be included if they had a stroke and want to improve their gait performance. To minimize the chance that improvements occur as a result of spontaneous recovery, only participants who are in the chronic stage of recovery (>6 months after stroke) will be included in the study. To prevent a ceiling effect, people with a low self-selected walking speed (<1.0 m/s) will be invited to participate. Finally, all people should be able to communicate in Dutch and complete a 3-stage command. People are excluded if they are unable to walk a minimum distance of 10 m; have a functional ambulation category score <3; have additional impairments not related to stroke, which influence their gait pattern, for example, severe osteoarthritis or amputation of the lower limb; have additional neurological impairments, for example, Parkinson disease that (severely) influence their walking performance.

### Sample Size

The sample size calculation is based on a randomized controlled trial with equal group sizes and “walking speed” (10-meter walking test, 10MWT) as a primary outcome measure [30]. The power is set at beta=.80, the significance level at alpha=.05, and a standard deviation of 0.23 m/s [31]. To demonstrate a significant change in walking speed, the minimal clinically important difference (MCID) is used and set at a minimum change of 0.16 m/s [32,33]. The calculations resulted in a minimum group size of 33 participants per group. Taking into account that 20% (7/33) of participants may be lost during (dropout) and after the intervention (loss to follow-up), this study aims to recruit 40 participants per group.

### Randomization, Blinding, and Treatment Allocation

#### Randomization Procedure

The allocation of participants to the experimental or the control condition will occur based on a computerized randomization program. Block randomization was calculated in block sizes of fours and sixes. The randomization procedure and the randomization scheme will only be available to an independent researcher who will not be involved in the delivery of the interventions or the performance of the measurements.

#### Blinding

The trained assessors are blind for treatment allocation. The therapists are aware of the treatment condition as they provide the explicit or implicit motor learning condition. The participants will probably also be aware of the treatment they receive; however, they will not be specifically told. The participants will be asked at each assessment not to reveal the details of treatment they received to the blinded assessor.

#### Training of Therapists and Treatment of Participants

**Figure 1:**
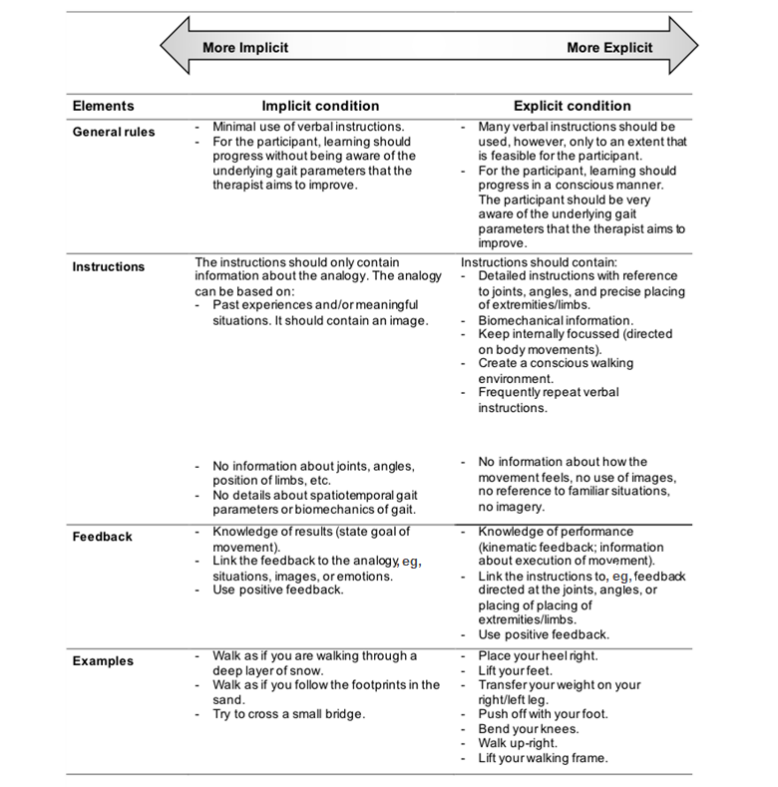
Characteristics of the interventions.

An intervention guideline is developed that outlines how the treatments (implicit and explicit conditions) should be delivered. The main aim of the intervention is to improve the quality of walking performance in people after stroke. The basic principle of the intervention guideline is based on the definitions of implicit and explicit motor learning by Kleynen et al [11]. For the purpose of this study, we strive to create the largest contrast between the conditions as possible. The main characteristics and differences in instructions, and feedback of the practice between interventions are described in Figure 1. Both conditions will always be applied to an extent that is feasible for the participant, and training will therefore always be tailored to the participant’s abilities within the given boundaries of the condition. Similar situations will be adopted in both conditions with regard to the “organization” of the training, for example, use variation in the (analogy) instructions and practice of the motor skill.

**Figure 2:**
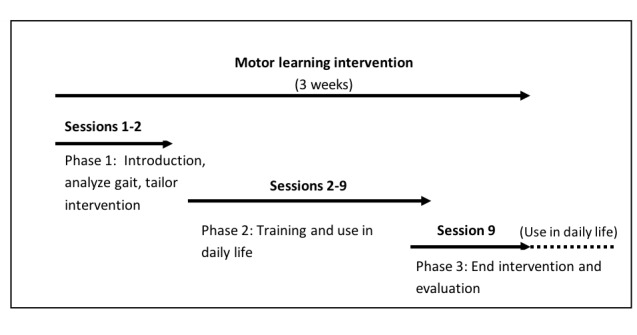
Overview of the gait training sessions.

The chosen intervention period was based on a preliminary study of Kleynen et al [28] that demonstrated that 3 weeks was a feasible period to develop and practice analogies with people after stroke. All participants will receive 9 training sessions in a 3-week intervention period, that is, 3 training sessions per week (Figure 2). Each training session takes place at the home of the participants and lasts for 30 min. Participants were asked to use the instructions in daily life (unguided therapy) and after the 3-week intervention period. To standardize the training content as much as possible, the therapists involved in the study will be trained before the start of the study. During 5 standardization training sessions, the intervention guideline will be discussed, explicated, and the therapists will be trained with the help of example cases. During the trial, therapists will attend 3 evaluation sessions to discuss the progress of the study and possible cases or difficulties they may experience during the intervention.

### Implicit Condition

Within the implicit condition, the concept of analogy learning is the leading approach as it has shown to adopt characteristics of implicit motor learning [24,27,28] and provides therapists with a practical and feasible guideline to organize gait training. To choose and develop appropriate analogies, the same procedure as used by Kleynen et al is followed [28]. A booklet with examples of possible analogies will be available for the therapists and patients as a source of inspiration. Preferably, analogies are developed based on the participants’ experiences and background to promote the personalized and meaningful aspects of analogy learning [28]. All analogy instructions used in the trial will be documented. In addition to analogy learning, the therapists are allowed to use elements based on the characteristics of implicit motor learning as reported under the implicit column in Figure 1.

### Explicit Condition

Within the explicit condition, gait training is organized by creating a learning environment that is (more) explicit in nature. Practice will be organized based on the characteristics of explicit motor learning as reported under the explicit condition in Figure 1. The condition is similar to the first (cognitive) stage of motor learning according to Fitts and Posner [34] that is characterized by the use of many explicit instructions, explaining precisely how motor skills should be performed. This stage is verbal and cognitive of nature. Contrary to the implicit condition, the explicit condition strives to maximize the number of explicit verbal instructions. The explicit instructions that have been used in the trial will be documented by the therapist’s in treatment logs.

### Measurements

All measures will be assessed by independent, blinded, and trained assessors at 3 assessment points (T0, T1, and T2; Table 1) and will take place at the participant’s home. The primary outcome parameter is walking speed measured in meters per second using 10MWT. First, demographics are described, and then, the primary and secondary outcome measures are reported.

### Demographics

At baseline, the following demographic and prognostic information is collected (T0): age, gender, time post stroke, affected side, walking aids, educational level, cognitive level (Montreal Cognitive Assessment, MoCA) [35], static balance and fall risk (Berg Balance Scale) [36], measures of mobility and disability (Rivermead Mobility Index) [37], and ability to make movements outside the synergetic patterns (Fugl-Meyer assessment of the lower limb) [38]. To assess the propensity for conscious motor processing, the Dutch version of Movement Specific Reinvestment Scale (MSRS) is used [39,40].

### Walking Performance Measures

Walking performance is measured using the 10MWT [41] and the Dynamic Gait Index (DGI) [42]. To assess the robustness of the obtained performance, walking will also be assessed over a longer period (1-month follow-up) and under secondary task loading.

#### 10-Meter Walking Test

Gain in walking speed has been associated with a transition to a higher class of ambulation, resulting in a better function and quality of life [41,43]. Next to statistical significance, the MCID will be used to assess clinical relevant differences. The MCID for walking speed in people after stroke has been established at the minimal difference of 0.16 m/s [33]. Exceeding this threshold indicates that the participants obtained a clinically meaningful improvement.

**Table 1:** Overview of measurements used in this study.

Data		Time	ICF^a^ level
**Demographics**			
	Age, gender, time post stroke, affected or nonaffected side, walking aids, educational level	T0	Personal factors
	Montreal Cognitive Assessment	T0	Body functions and structure
	Berg Balance Scale	T0	Activity level
	Rivermead Mobility Index	T0	Activity level
	Fugl-Meyer Assessment	T0	Body functions and structure
**Walking performance**			
	10-meter walking test	T0, T1, T2	Activity level
	Dynamic Gait Index	T0, T1, T2	Activity level
	Dual task	T0, T1, T2	Activity level
**General outcome measures**			
	Movement Specific Reinvestment Scale	T0, T1, T2	Personal factors
	Verbal protocol	T2	N/A^b^
	Stroke Specific Quality of Life Scale	T0, T2	Participation level
	Global Perceived Effect scale	T2	N/A

^a^ICF: International Classification of Functioning, Disability, and Health.

^b^N/A: not applicable. The ICF level is not applicable for the Verbal Protocol and Global Perceived Effect Scale as these measures do not examine health or disability but evaluate the intervention.

#### Dynamic Gait Index

The DGI is a physical performance test that assesses the gait, balance, and fall risk and has shown to have a good reliability and validity in people after stroke [44,45]. Eight different tasks related to the balance and gait, for example, walking, turning, and stepping over objects are assessed [46]. The performance will be scored according to the modified DGI as proposed by Shumway and Cook as the extended scoring system has shown to possess good psychometric properties [42,47].

#### Dual Task

In this study, people will be asked to complete a tone-counting task similar to that proposed by Wilson et al [48]. In this task, people will be exposed to 4 different sounds (buzzer, ping, tone and bell ring) in a randomized order over a 30-s time period. They will be asked to only count a specific target sound (eg, bell ring) and ignore the other 3 distracting sounds. The task will be performed twice, once as a single task and once while walking concurrently (dual task). The actual number of tones will be compared with the estimate number of tones by the participants. Error scores (actual minus estimate) will be calculated as a measure for the dual-task performance.

### Self-Reported Measures

#### Movement-Specific Reinvestment Scale Adapted for Gait

The MSRS is a questionnaire that measures a person’s inclination for conscious control. People after stroke have shown to have greater propensity to conscious processing compared with the age-matched, nondisabled population [49,50]. In this study, an adapted version of the MSRS specific to gait is used. Adapted versions of the MSRS have been used before, for example, for putting movements in golf [51,52]. The MSRS contains one factor related to conscious control (conscious motor processing) and one related to self-consciousness about movement (movement self-consciousness). Each factor of the MSRS comprises 5 statements, such as “I try to think about my movements when walking” (conscious motor processing) and “I am concerned about what people think about me when I am walking” (movement self-consciousness). The statements will be assessed with binary response (yes/no) [40]. The MSRS will be measured at baseline (T0) to describe the population and over time (T0, T1, and T2) to assess how much gait-related conscious processing takes place.

#### Verbal Protocol

To assess the amount of explicit knowledge, a verbal protocol questionnaire as used in Orrel et al will be administered after the 3-week intervention [22]. Explicit knowledge is assessed by examining the number of explicit rules that the participant uses during walking. Participants will be asked to report any “rules, methods, or techniques” that they have thought about or used and that have improved or impaired their walking performance. A rule is defined as any statement containing at least one of the following aspects: a movement or position of one limb, a movement or position of one joint, a velocity of a limb movement, an angle or direction of a joint or the spine, or the placement of the walking aid. Each statement containing a single limb, joint, or other body part will be counted as 1 rule. If a statement contains 2 (or more) different limbs, joint and body parts, or different directions or angles, they are counted separately (eg, “I tried to lift my foot and put it more forward.”). Statements are excluded if they are irrelevant to walking performance or do not refer to technical aspects about walking (eg, “More concentration needed.”). The answer to the verbal protocol will be screened by 2 independent researchers who will be blind to the experimental condition. Their agreement will be investigated using a correlation coefficient (or ICC).

#### Stroke and Aphasia Quality of Life Scale

The *Stroke and Aphasia Quality of Life Scale (* SAQOL-39) is assessed at baseline and after the intervention to measure the health-related quality of life [53]. The questionnaire contains 39 items and is developed for people after stroke and is feasible to use for people with aphasia. The SAQOL-39 is a short version of the original SAQOL (53 items) and has shown to be an acceptable, reliable, and valid measure of the health-related quality of life [53].

#### Global Perceived Effect

The *Global Perceived Effect* scale is a reliable method to assess the participant’s satisfaction and will be used to evaluate the participant’s perception of the intervention [54]. The Global Perceived Effect scale will involve the following question: To which extent did your walking ability change over the last three weeks? The question will be scored on a 7-point Likert scale from “completely improved” to “completely deteriorated.”

### Process Evaluation

To gain an insight into the process-related factors that may have influenced the effectiveness of the 3-week analogy learning walking intervention, a process evaluation will take place along the study [55]. Data will be collected to (1) asses to what extent the therapists delivered the interventions as intended (fidelity), (2) explore the therapists’ opinions and experiences about the interventions with regard to the feasibility and possible effects, and (3) explore the patients’ opinions and experiences about the interventions with regard to the feasibility. Table 2 represents the data collection methods used to assess the different aspects of the process evaluation. For both groups, the provided instructions will be documented and evaluated in the therapists’ logs. In addition, short questionnaires will be used to administer the therapists’ and participants’ opinions about the gait training after the completion of the 3-week intervention. To explore the extent to which the interventions were implicit or explicit in nature, the verbal protocol will be assessed. Evidence indicates that implicit learning is typically characterized by the less accumulation of explicit rules compared with explicit learning [24,56].

To monitor the integrity of the intervention, self-reported (subjective) and audio-recorded (objective) data will be evaluated. First, all therapists are required to self-report any deviations from the treatment protocol or other incidents during the session in a log after every session. All instructions used during therapy sessions will be recorded in the therapists’ and patients’ logs. Furthermore, patients can use the log to write down any possible events that might have occurred during unguided therapy. The patient log is, therefore, only used as a reminder for unguided therapy and as a communication tool between therapists and participants. Second, 10 gait trainings will be randomly selected (5 implicit and 5 explicit interventions) and audio recorded. Both self-reported and audio-recorded data will be screened to evaluate whether the intervention was delivered according to the protocol.

### Data Analyses

Baseline scores of demographic and prognostic data and primary and secondary outcome measures will be used to compare the 2 groups. Only data of the participants who attended minimum of 7 or more of the therapy sessions will be considered as adherent and processed in the statistical analysis. Statistical analysis will be conducted to determine significant differences between groups and over time (baseline and postintervention performance). A repeated measure analyses will be used to compare the 2 groups (implicit and explicit) at 3 different time points (before, after, and after 1-month follow-up). Post hoc tests with correction for multiple testing will be used to explore effects over time and between groups. Subgroup analysis will be performed on cognition (MoCA score <21) [35]. An alpha level of .05 will be set for all tests. The primary outcome measure, walking speed, will also be described with reference to clinically relevant differences between groups (MCID: 0.16 m/s) [33]. All datasets used or analyzed during this study are available from the corresponding author on a reasonable request.

Data will be analyzed according to an intention-to-treat and per-protocol principle. In the intention-to-treat, data of the participants are analyzed according to their original treatment allocation. If self-reported (subjective) and audio-recorded (objective) data reveal that cases are not delivered as intended, then the analyses will be performed using the per-protocol principle. Within this study, protocol deviations are defined as “deviations from the protocol that occur in two or more sessions.” If protocol deviations were observed, then data from this person were not included in the per-protocol analysis. Data in the process evaluation related to the therapists’ and patients’ opinions and experiences toward the feasibility of the intervention and perceived benefits will be analyzed by means of descriptive statistics. Free comments and clarifying examples may be quoted and used to describe personal experiences of the therapists or participants.

**Table 2:** Measures for the process evaluation. A checkmark indicates with which measure the question is examined.

Questions	Measures			
	Therapist Log	Audio recordings	Therapist questionnaire	Patient questionnaire
To what extent did the therapists deliver the interventions as intended (fidelity)?	✓	✓	✓	
How did the therapist’s experience delivering the interventions with regard to the feasibility and possible effects?			✓	
How did the patients’ experience the intervention with regard to the feasibility?				✓

## Results

The entire project was funded in September 2015. Patient enrolment began in March 2017 and is expected to continue until July 2018. Following completion of data collection, data cleaning and analyses will take place. The first study results are expected to be submitted for publication in 2019.

## Discussion

In this paper, we described the methodology of a randomized controlled single-blinded study that evaluates the potential effects of implicit motor learning compared with explicit motor learning in the gait rehabilitation of people suffering from stroke. The relevance of the study and the advantages and disadvantages of several aspects of the chosen design are discussed.

### From Laboratory Setting to Clinical Practice

Although motor learning research is growing exponentially, most published studies have been performed in laboratory settings. However, it is important to understand the application of motor learning within clinically relevant environments and the influence of interventions on the completion of everyday functional tasks [18,57]. A recent systematic review on implicit motor learning in people after stroke pointed out that studies performed within clinical settings are limited [18]. Of the 20 included studies, only 1 study involved a clinically relevant task [22]. To improve the generalizability of research findings toward clinical practice and to the broader population, various choices on different aspects in the research design had to be made. First, with regard to the task and environment, this study involves gait training that takes place at the homes of the participants. Gait is a functional daily life motor skill, and it is advised to organize practice in a context-specific environment [4,58]. Therefore, compared with the current state of evidence, this study adds insights into the effects of motor learning in a clinically relevant environment and for a clinically relevant task.

Second, in this study, the motor learning conditions are tailored to the individual participants. Most implicit motor learning studies use strict research protocols in which each participant usually receives the exact same instructions (eg, the whole experimental group receives the same right-angled triangle analogy to learn topspin forehand in table tennis), whereas the explicit learning is promoted by using the same set of verbal instructions [24]. A one-size-fits-all approach may not be ideal for the clinical population as they generally demonstrate a large variety in degree and types of impairments. The use of personalized analogies allows the physiotherapists to respond to the individual walking impairments and emphasize on the meaningful component of analogy learning. With a personalized approach (more comparable with the real-life practice), the instructions may be less standardized. Therefore, steps were undertaken to ensure the quality of the interventions. Before the study, all therapists were familiarized and trained with the implicit and explicit conditions. Measures (therapists’ logs and audio recordings of the interventions) were selected to evaluate whether the trainings were delivered as intended, and data will be analyzed as per the intention-to-treat and per-protocol (see the Data Analyses section).

### Methodological Aspects

Within this study, extra attention was given to the following 3 methodological aspects in designing the intervention: contrast, content of the interventions, and target population. First of all, it is important to address that in many rehabilitation studies, the contrast between the intervention and control group (generally usual care only) turns out to be too modest, which therefore results in neutral study results [59]. The underlying reason may be that rehabilitation interventions are often too complex to control for influences of other interventions and that the control intervention group (therapy as usual) is often poorly described. We tried to overcome these problems by including participants in the chronic phase of recovery, who do not receive additional interventions directed at the improvement of gait. Furthermore, we strive to ensure the contrast of the interventions by using a guideline, written protocol. In this protocol, the delivery of both conditions is clearly defined. It transparently describes how the 2 interventions differentiate from each other. To ensure the integrity of the intervention, the logs and audio-recorded therapy sessions will be conducted and evaluated.

Another decision we would like to address is the choice for the interventions. In clinical practice, often mixtures of implicit and explicit forms of learning are used or therapists switch between different forms of learning [15,60]. However, it remains unclear whether mixing implicit and explicit learning or switching between the forms is effective and/or necessary. The current design is necessary to evaluate the effects of implicit versus explicit learning in clinical practice, and so we believe that it is (ethically) legitimate to compare 2 distinct interventions and not use “therapy as usual” as a control intervention. The interventions in this study will be delivered in a personalized manner regarding overall components of the interventions, for example, the sort of gait impairments, amount of repetitions, and use of personalized analogies. However, the content of the interventions applied is clearly outlined in the guidelines, and therapists are required to strictly follow these.

Furthermore, within the target population, people experiencing cognitive impairments or communicative restrictions are often excluded. In this study, we strive to include a sample that reflects the broad range of impairments reported after stroke. In earlier studies, it has been shown that motor learning interventions might be effective, also for people with cognitive and/or communicative impairments [27,28].

### Implicit Motor Learning

Scientific evidence describes that implicit learning is typically characterized by robust dual-task performance, durable performance over time, and less accumulation of explicit rules [24,56]. The dual-task measure and the 1-month follow-up session were specifically chosen with respect to these implicit characteristics. First, walking while concurrently carrying out a secondary (tone counting) task [48] places high information-processing demands on the learners. In contrast to explicit learners, implicit learners showed that performance was not disrupted in dual-task conditions, which indicates that they may be able to free up attentional resources to perform the secondary task [24]. We therefore predict that the implicit condition will remain stable under secondary task burden. For this reason, implicit learning may be particularly beneficial for those who experience cognitive impairments that are commonly seen in people after stroke.

Then, a recent study by Tse et al [25] found performance improvements after a 2-day separation; however, they recommended to include a longer separation to test skill consolidation. This study will therefore evaluate performance after the 1-month follow-up period. It is hypothesized that performance improvements in the implicit group will remain robust over a longer period (1-month follow-up). To make statements about long-term effects within rehabilitation, it may be desirable for future studies to include even longer follow-up periods (>3 months). Finally, a verbal protocol will be assessed because it is hypothesized that participants within the implicit learning group will report fewer explicit rules than the control group, which would be in line with the findings of earlier studies [23,24]. Even though the starting point of the explicit intervention is to use many verbal explicit instructions and provide more details on the motor performance, the exact number of explicit rules will be tailored to the ability of the patient to process these rules. Therefore, in practice, some patients might receive higher numbers of explicit rules than others. Still, we hypothesize that the explicit group will require more explicit rules compared with the implicit group.

### Conclusion and Implementation

With the description of the study design, we hope to contribute to the discussion on how a tailored but standardized form of implicit motor learning could be applied in clinical practice. The relevance of the study and the advantages and disadvantages of several aspects of the chosen design are discussed (eg, personalized approach, sample selection).

## Abbreviations

10MWT: 10-meter walking test
DGI: Dynamic Gait Index
MCID: minimal clinically important difference
MSRS: Movement Specific Reinvestment Scale
SAQOL-39-NL: Stroke and Aphasia Quality Of Life

## Appendix

Multimedia Appendix 1 Peer review report by Dutch sponsor, Stichting Innovatie Alliantie (Innovation Alliance Foundation).
