# Supplementary material for: The Effects of Implicit and Explicit Motor Learning in Gait Rehabilitation of People After Stroke: Protocol for a Randomized Controlled Trial
Source: JMIR Res Protoc. 2018 May 24;7(5):e142. doi: 10.2196/resprot.9595 (PMC5992456; doi:10.2196/resprot.9595)
Supplement: Multimedia Appendix 1 [file resprot_v7i5e142_app1.pdf]

ZU
YD

De kracht van het onbewuste 2.0

RAAK-PRO – reactie op advies commissie en daarbij geplaatste opmerkingen
Registratienummer: 2014-01-49PRO
Projectleider: Dr. Susy Braun

Het consortium van de RAAK PRO aanvraag "De kracht van het onbewuste 2.0" is verheugd dat de commissie een positief advies heeft gegeven t.a.v. onze aanvraag. We zijn er dan ook van overtuigd dat we met dit onderzoek een belangrijk onderwerp in de zorg adresseren en dat het onderzoek leidt tot relevante toepassingen voor professionals en cliënten. Het positieve advies en de beoordeling van de commissie van de vraagarticulatie, netwerkvorming en onderzoeksplan sterkt onze overtuiging.

In haar reactie vraagt de beoordelingscommissie m.b.t. het onderzoeksplan aandacht voor twee punten. De genoemde aandachtspunten zijn:

- *De grote diversiteit binnen de CVA-patiëntenpopulatie: de commissie geeft mee dat het belangrijk is de verschillende patiëntengroepen (bv. patiënten met een cognitieve stoornis versus patiënten met motorische problemen) duidelijk te definiëren ten opzichte van het doel van het onderzoek.*
- *Het inzetten van een controlegroep om de effecten te meten van het inzetten van de beeldwatch.*

Op beide aandachtspunten willen wij graag reageren.

Reactie m.b.t. de opmerking over de diversiteit van de CVA-patiëntenpopulatie:
Doel van het onderzoek is om de potentie van onbewust motorisch leren te onderzoeken bij mensen die na een beroerte 'loop'-problemen ervaren. Op basis van de bestaande evidence in de literatuur zouden cliënten na een beroerte baat kunnen hebben bij deze vorm van leren. De meerwaarde van impliciet motorisch leren zal waarschijnlijk (relatief) groter zijn voor mensen die naast fysieke ook cognitieve problemen hebben[1,2]. Cognitieve stoornissen komen bij een grote groep cliënten binnen de beroertepopulatie voor (>60%)[3].
De brede inclusiecriteria en daarmee verbonden diversiteit zijn daarom een weloverwogen keuze. Bovendien blijven we zo dicht bij de praktijk waar deze grote variatie aan cliënten gezien wordt.
Alle te includeren deelnemers moeten dus loopproblemen ervaren en een meetbare loopafwijking (< 1,0 m/s) hebben[4]. Om achteraf te kunnen bepalen welke groepen cliënten het meeste baat hebben bij welke vorm van motorisch leren worden naast deze gegevens ook andere factoren die van invloed kunnen zijn op de uitkomsten in kaart gebracht. Aan de reeds in het protocol beschreven factoren worden daarvoor de volgende data nog toegevoegd: score neuropsychologische screening[5] op geheugen, aandacht en informatieverwerking evenals lokalisatie van het CVA.

Referenties

1. Steenbergen B, van der Kamp J, Verneau M, Jongbloed-Pereboom M, Masters RS. Implicit and explicit learning: applications from basic research to sports for individuals with impaired movement dynamics. Disabil Rehabil. 2010;32(18):1509-16.
2. Maxwell JP, Masters RS, Eves FF. The role of working memory in motor learning and performance. Conscious Cognition. 2003;12(3):376-402.
3. Rasquin SM[1], Lodder J, Ponds RW, Winkens I, Jolles J, Verhey FR. Cognitive functioning after stroke: a one-year follow-up study. Dement Geriatr Cogn Disord. 2004;18(2):138-44. Epub 2004 Jun 18.
4. Combs-Miller SA, Kalpathi Parameswaran A, Colburn D, Ertel T, Harmeyer A, Tucker L, et al. Body weight-supported treadmill training vs. overground walking training for persons with chronic stroke: a pilot randomized controlled trial. Clin Rehabil. 2014;28(9):873-84.
5. Nederlands Instituut van Psychologen. Richtlijn voor een kort neurologisch onderzoek bij patiënten met een beroerte. 2010

Reactie m.b.t. de opmerking over de controlegroep (werkpakket C):
In de aanvraag is in werkpakket C gekozen voor een cohortstudie omdat de focus van dit werkpakket ligt op de hanteerbaarheid en de acceptatie van de technologieën. Bij mensen na een beroerte is vrijwel geen onderzoek naar technologie-acceptatie uitgevoerd[1]. Het is het daarom onduidelijk welke factoren het al dan niet gebruiken van technologie in deze doelgroep beïnvloeden[2]. Ook is er weinig bekend over de effectiviteit van deze eenvoudige technologieën op 'het lopen' bij mensen na een beroerte[3,4].
Wij zijn het eens met de commissie dat toevoeging van een controlegroep meer inzicht zou geven in mogelijke effecten van de technologieën op het lopen. We willen daarom voorstellen om de interventiegroep van werkpakket B (n=40) voor start van werkpakket C te randomiseren in 2 groepen, waarbij 1 groep de technologie krijgt en de andere als controlegroep fungeert. De mate van oefenen wordt in beide groepen geregistreerd. Na een maand krijgt de controlegroep alsnog de technologie. Door dit design blijft de gehele groep voor de hanteerbaarheids- en acceptatievragen bewaard en wordt er tevens inzicht in eerste effecten verkregen. Op basis van studies met vergelijkbare groepsgrootte naar technologieën in andere populaties, lijkt het mogelijk om met deze aantallen (significante) verschillen te vinden[5,6].

Referenties

1. Chiu TM, Eysenbach G. Stages of use: consideration, initiation, utilization, and outcomes of an internet-mediated intervention. BMC Med Inform Decis Mak. 2010 Nov 23;10:73.
2. Peek ST, Wouters EJ, van Hoof J, Luijkx KG, Boeije HR, Vrijhoef HJ. Factors influencing acceptance of technology for aging in place: a systematic review. Int J Med Inform. 2014;83(4):235-48.
3. Shull PB, Jirattigalachote W, Hunt MA, Cutkosky MR, Delp SL. Quantified self and human movement: a review on the clinical impact of wearable sensing and feedback for gait analysis and intervention. Gait Posture. 2014;40(1):11-9.
4. Casamassima F, Ferrari A, Milosevic B, Ginis P, Farella E, Rocchi L. A wearable system for gait training in subjects with Parkinson's disease. Sensors. 2014;14(4):6229-46.
5. Nanhoe-Mahabier W1, Allum JH, Pasman EP, Overeem S, Bloem BR. The effects of vibrotactile biofeedback training on trunk sway in Parkinson's disease patients. Parkinsonism Relat Disord. 2012 Nov;18(9):1017-21.
6. Schwenk M, Grewal GS, Honarvar B, Schwenk S, Mohler J, Khalsa DS, Najafi B. Interactive balance training integrating sensor-based visual feedback of movement performance: a pilot study in older adults. J Neuroeng Rehabil. 2014 Dec 13;11(1):164.

Heerlen, 7 april 2015

Mede namens de consortiumleden en projectdeelnemers
Dr. Susy Braun
Projectleider
